# Delineating Areas of Past Environmental Degradation near Smelters using Rock Coatings: A Case Study at Rouyn-Noranda, Quebec

**DOI:** 10.1038/s41598-018-35742-4

**Published:** 2018-11-26

**Authors:** David W. Leverington, Michael Schindler

**Affiliations:** 1 Department of Geosciences, Texas Tech University, Lubbock, Texas 79409 USA; 2 Harquail School of Earth Sciences, Laurentian University, Sudbury, Ontario P3E 2C6 Canada

## Abstract

Emissions of SO_2_ from smelters can promote formation of acid rain, which can dissolve siliceous minerals on exposed rock surfaces and promote the formation of silica gel layers within which detrital and smelter-derived particulates can become trapped. These processes of dissolution and entrapment can result in the formation of rock coatings that contain elevated levels of heavy metals. Between 1927 and 1976, the Horne smelter processed sulfide ore derived from the Rouyn-Noranda region and became one of the largest emitters of particulates and sulfur dioxide in North America, promoting the formation of coatings on nearby rock surfaces. The reflectance spectra of these coatings are relatively flat, with typical reflectance values ranging between ~5% at visible wavelengths and ~16% in the shortwave infrared. Absorption troughs in coating spectra are consistent with the presence of materials including opaline silica, olivine, pyroxene, hydrous phyllosilicates, and sulfates. Classification of Landsat 8 Operational Land Imager data indicates that rock coatings near Rouyn-Noranda comprise a total surface area of ~1.5 km^2^, nearly all of which is located within ~6 km of the Horne smelter. Remote sensing techniques can used to delineate the geographic extents of coatings near smelters, highlighting areas previously subjected to severe environmental degradation.

## Introduction

The smelting of sulfide ores can produce atmospheric emissions rich in particulates and heavy metals^1,2^, resulting in contamination of nearby soils, vegetation, and water bodies^3,4^. Also, emissions of sulfur dioxide from smelters can promote formation of acid rain, which along with other types of emissions can impact local vegetation health and reduce vegetation cover^5,6^. Under extreme conditions, acid rain can dissolve siliceous minerals on exposed rock surfaces and thereby promote the formation of thin silica gel layers within which detrital and smelter particulates can become trapped^7–9^. Over time, these processes of dissolution and entrapment can result in the formation of brownish-black rock coatings that contain components such as oxides, sulfates, high-temperature silicates, carbon-rich particulates, and rock and soil particulates; and that are characterized by high levels of heavy metals^10^.

The use of remote sensing techniques in the detection and mapping of rock coatings near smelters can help to delineate the geographic extents of areas subjected to past environmental degradation, and has the potential to be applied toward the monitoring of ongoing degradation at sites where emissions continue to promote the development of coatings^11,12^. Related techniques may also prove useful in the monitoring of environmental recovery where smelter emissions have been reduced. In prior work, the supervised classification of Landsat Enhanced Thematic Mapper Plus (ETM+) and Hyperion satellite images was successfully used to determine the spatial distribution of rock coatings near three smelting sites in the Sudbury region of Ontario, Canada, highlighting areas previously exposed to relatively high levels of acid rain and smelter-derived particulates^11,12^.

**Figure 1:**
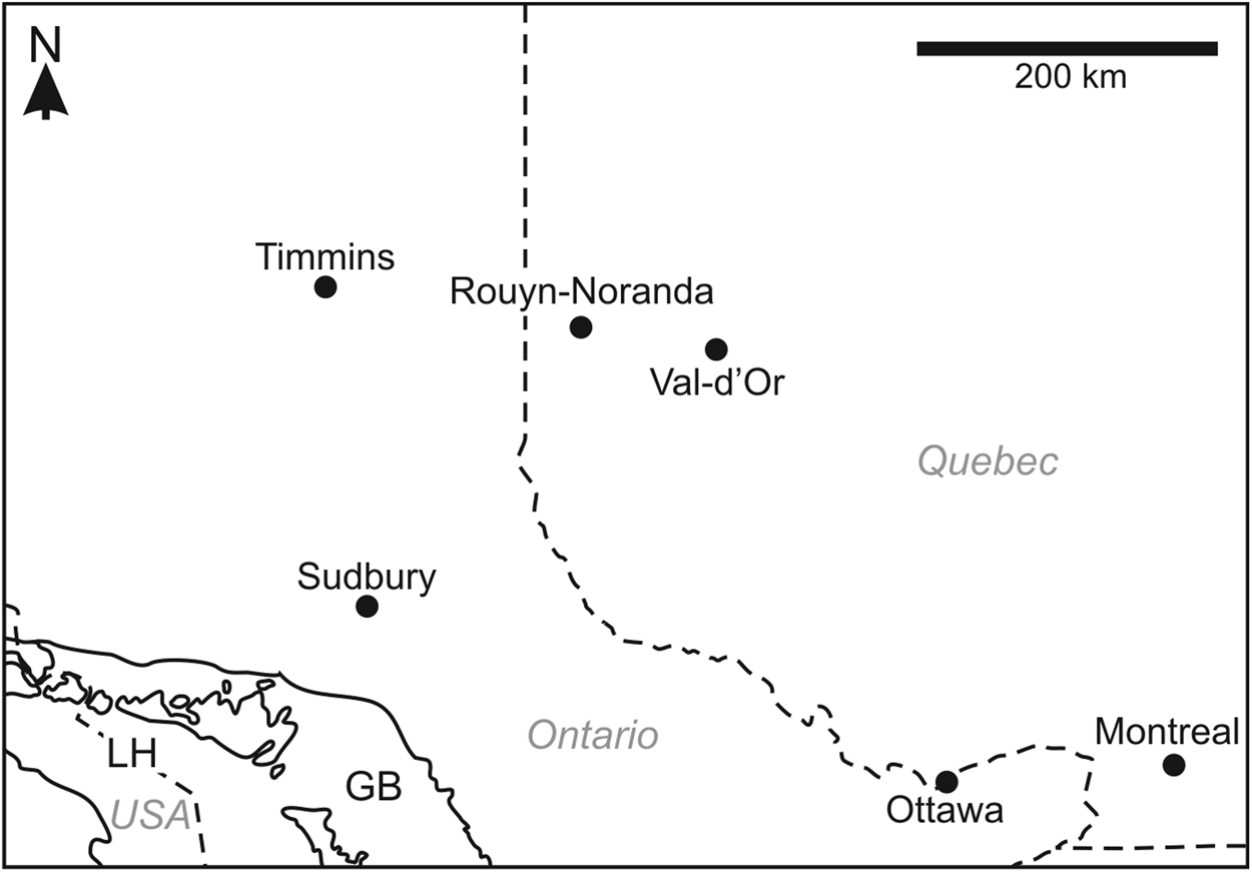
Rouyn-Noranda is located in western Quebec, Canada, between the cities of Val-d’Or, Quebec, and Timmins, Ontario. These three cities all fall within the Abitibi gold belt, a major mining district within the Abitibi greenstone belt. The Horne smelter is located in Rouyn-Noranda, and the surrounding region hosts some of the largest volcanic massive sulfide deposits in the world. Labelled water bodies: Lake Huron (LH) and Georgian Bay (GB).

This study extended the scope of earlier work at Sudbury, Ontario, to the Rouyn-Noranda region of Quebec (Fig. 1), in order to further evaluate remote sensing techniques in the detection, mapping, and characterization of rock coatings near smelters. Between 1927 and 1976, the Horne smelter processed sulfide ore derived from the Rouyn-Noranda region and ultimately became one of the largest emitters of particulates and sulfur dioxide in North America^13,14^, negatively affecting local vegetation vigor and extent^15,16^ and contaminating surface materials with heavy metals^17–19^. Over time, coatings formed on exposed rock surfaces proximal to the Horne smelter^20^.

In this study, the reflectance spectra of rock coatings from the Rouyn-Noranda region were measured and described, and were compared with those previously collected for the Sudbury region. Also, a reflectance database derived from Landsat 8 Operational Land Imager (OLI) data for this region was classified in order to determine the spatial distribution of rock coatings produced by the Horne smelter, and to further evaluate the utility of remote sensing techniques in the study of environmental degradation caused by smelters.

## The Rouyn-Noranda Study Area

**Figure 2:**
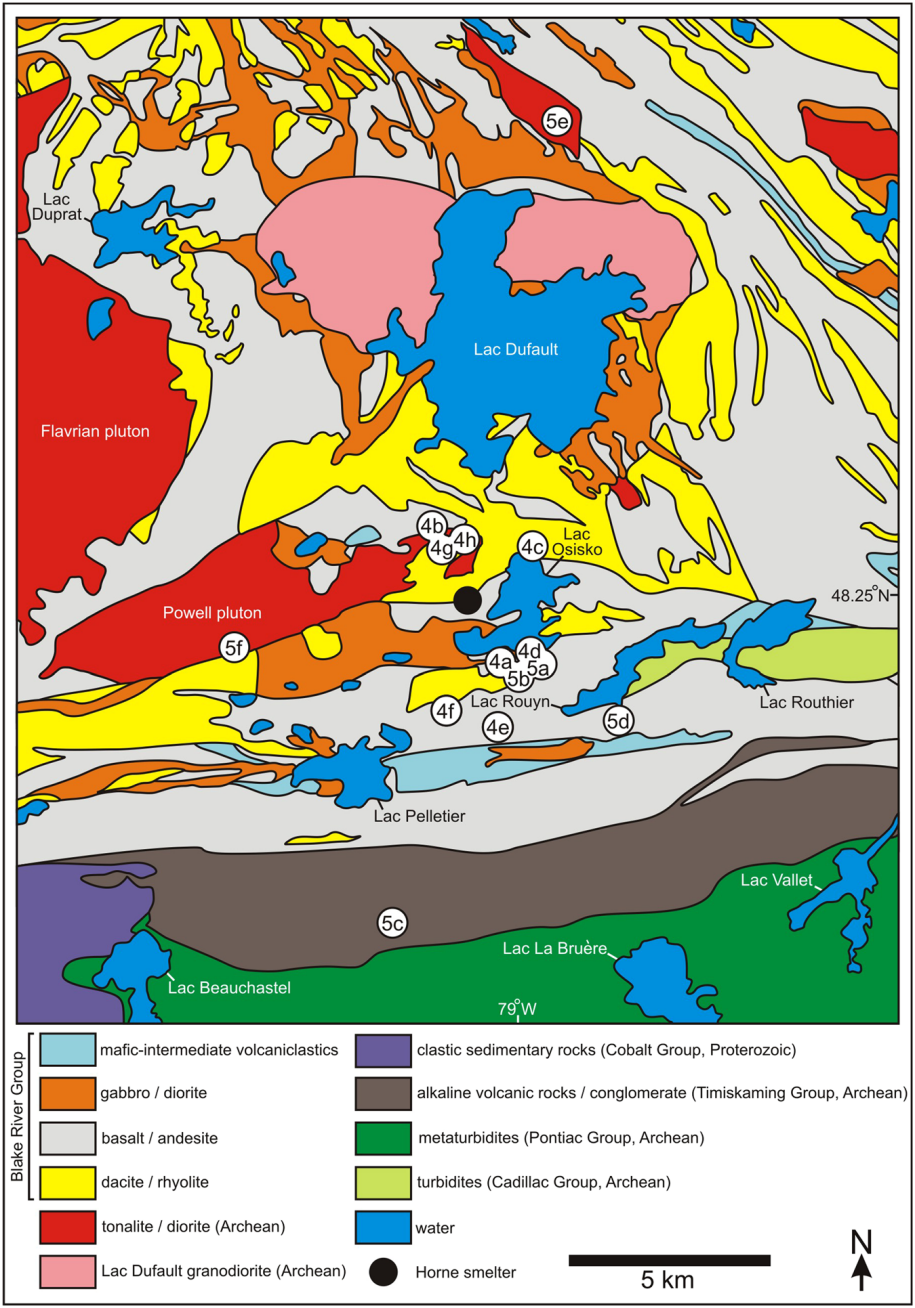
Bedrock geology of the Rouyn-Noranda study area^24^. The northern and central parts of the study area are dominated by Archean volcanic units of the Blake River Group, which is a southern unit of the Superior Province’s Abitibi greenstone belt. The southern part of the Rouyn-Noranda study area is partly comprised of predominantly sedimentary units of the Timiskaming Group, the Cadillac Group, the Cobalt Group, and the Pontiac Group. The location of the Horne smelter is given by a black circle at lower center, immediately west of Lac Osisko. The locations of sites depicted in Figs 4 and 5 are identified by white circles and are labeled by figure number. Sites depicted in Fig. 3A,B are located in the same general area as given for Fig. 4H.

The Rouyn-Noranda study area is 35 km by 20 km in dimension and is approximately centered on the Horne smelter (Fig. 2), which is located on the western shores of Lac Osisko and within the city of Rouyn-Noranda, Quebec. Bedrock of the northern and central parts of the study area is dominated by Archean igneous materials of the Blake River Group, a southern unit of the Superior Province’s Abitibi greenstone belt^21,22^. Further to the south are predominantly sedimentary units of the Timiskaming and Cadillac groups (both also part of the Abitibi greenstone belt), the Cobalt Group (a Paleoproterozoic unit of the Huronian Supergroup), and the Pontiac Group (a northern unit of the Pontiac Subprovince)^23,24^ (Fig. 2). The Abitibi greenstone belt has mostly been subjected to low-grade metamorphism and predominantly displays greenschist to subgreenschist facies^25,26^. Cobalt Group materials within Quebec have similarly been subjected to low-grade metamorphism^27^, but the Pontiac Subprovince is of medium metamorphic grade near its northern boundary with the Abitibi greenstone belt^28^.

The 2.7 Ga Blake River Group mainly consists of volcanic units of basaltic to rhyolitic composition^26,29^. All associated lava flows are interpreted to have been emplaced in a submarine environment, based in part on the widespread occurrence of pillow lavas and pillow breccias (including hyaloclastites) and the presence of turbidite and argillite interbeds^26,30^. Volcanic units of the Blake River Group were intruded by several generations of plutons, dikes, and sills^26^, with the most prominent intrusions including the Flavrian and Powell tonalites and the Lac Dufault granodiorite^29,31^ (Fig. 2).

The Blake River Group hosts more than thirty volcanogenic massive sulfide (VMS) deposits^26,31^. In the Rouyn-Noranda region, these VMS orebodies are associated with rhyolite flows and felsic fragmental rocks of the Noranda volcanic complex^29,31–33^, a 35-km-diameter volcanic center composed of alternating mafic and felsic units that are crosscut by dikes of gabbroic and dioritic composition^34,35^. The related Horne deposit is one of the world’s largest VMS orebodies, having produced 260 tonnes of gold and 1.13 megatonnes of copper between 1927 and 1976^35^. Ore minerals associated with the Horne and nearby VMS deposits include pyrite, pyrrhotite, chalcopyrite, galena, sphalerite, and magnetite, as well as native gold and silver^32,36^.

The study area is located in the boreal ecoclimatic region of Canada^37^. Topographic relief here is less than 150 m, and bedrock exposure is discontinuous. Glacial lakes Ojibway and Agassiz-Ojibway extended across much of western Quebec in the Early Holocene, and glaciofluvial, glaciolacustrine, and glacial till deposits are variously distributed across parts of the study area^38,39^. The study area falls within the balsam fir and white birch domain of the boreal vegetative zone, with common tree types including white spruce, black spruce, balsam fir, aspen, birch, and jack pine^40^. Lichen cover can be extensive on local bedrock^41^.

### The Horne Smelter

**Figure 3:**
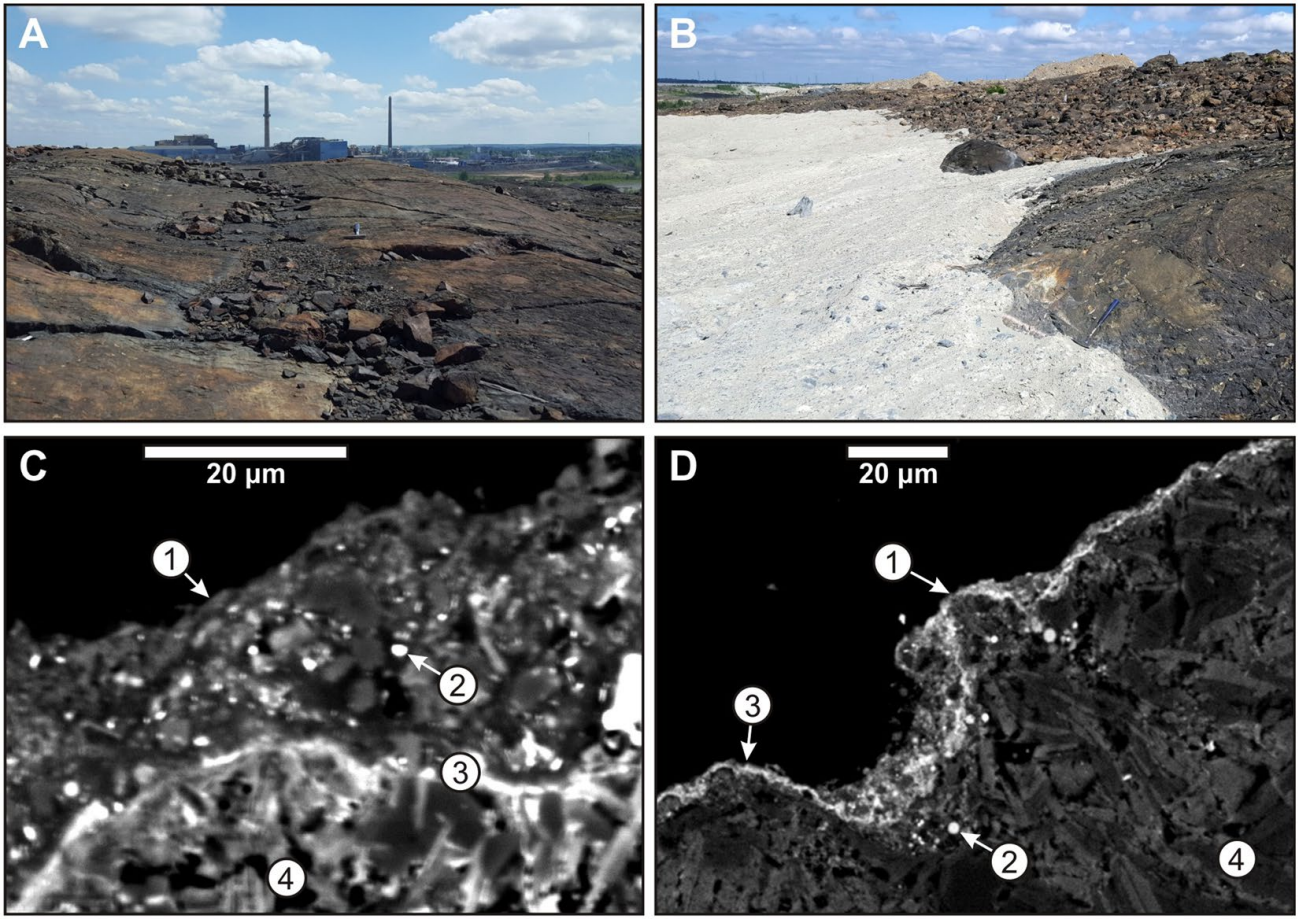
(**A**) Extensively coated igneous extrusives and intrusives of felsic composition are located immediately north of the Horne smelter. Rock hammer near photo center provides scale for foreground materials, and two smelter stacks are visible in the background. Site location is given in Fig. 2. (**B**) Site of partial burial of coated felsic bedrock located immediately north of the Horne smelter in Rouyn-Noranda, related to new road and infrastructure construction. Activities such as these continue to reduce the surface exposure of rock coatings in the Rouyn-Noranda region. Rock hammer in right-center foreground for scale. Site location is given in Fig. 2. (**C**,**D**) Scanning electron microscope (SEM) images of two rock coatings collected in the Rouyn-Noranda study area. (**C**) Coating on a basalt (coating contains 5 wt% Pb, 0.3 wt% As, and 0.7 wt% Cu; smelter distance = 4.0 km); (**D**) coating on a felsic igneous rock (coating contains 1.5 wt% Pb, 0.063 wt% As, and 0.75 wt% Cu; smelter distance = 1.2 km). Labeled features: (1) coating surfaces; (2) particulates (bright) within a silica-rich matrix (dark); (3) metal-rich sulfate layers; (4) rock substrates. The silica-rich matrix of local coatings typically contains a wide range of particulates including high-temperature silicates and oxides emitted by the Horne smelter, as well as detrital grains derived from nearby soils^20^. Metal-rich sulfate layers are commonly present along coating surfaces (**D**), in the interior of coatings (**C**), or along the interfaces between coatings and underlying substrates.

The Horne smelter (Figs 2 and 3A) processed local sulfide ore between 1927 and 1976, and during this time frame became one of the largest North American emitters of particulates and sulfur dioxide^13,14^. Metals released into the atmosphere during and following this period have most notably included lead, zinc, nickel, arsenic, copper, and cadmium^17,19,42–47^. Annual emission of particulate matter here peaked in 1965 at more than 1.5 million metric tonnes^13^. Pollution controls were implemented at the Horne smelter beginning in the 1970s, resulting in reductions in total emissions^14^. The Horne smelter ceased the processing of local sulfide ores in 1976, and over the following decades became a processor of electronic scrap containing copper and precious metals.

As with emissions of particulate matter, annual emissions of sulfur dioxide peaked in 1965 at about 710,000 metric tonnes, and have steadily declined since that time^48^. In areas proximal to the Horne smelter, such emissions have previously stunted the growth of trees^16^ and affected the abundances of some varieties of vegetation (e.g., reducing or eliminating the presence of *Sphagnum fuscum* peat moss^13,15,42^). Smelter emissions may have also increased the acidity of some lakes in the region^49^. Soils, peat hummocks, aquatic organisms, and seasonal snow in the Rouyn-Noranda region have contained elevated levels of heavy metals both during and following the period of peak annual emissions, with metal concentrations generally decreasing with increasing distance from smelter stacks^13,15,17–19,40,50,51^.

### Rock Coatings Near the Horne Smelter

Acid rain generated near the Horne smelter prior to the mid-1970s interacted with exposed rock surfaces to form thin silica gel layers, causing the entrapment of detrital and smelter particulates within gel layers and additionally promoting the formation of metal sulfate layers^20^. Over time, these processes promoted development of prominent rock coatings with colors that range between black and reddish-brown (Fig. 3A).

Rock coatings in the Rouyn-Noranda region are most commonly ~5 to 50 micrometers thick, and are predominantly composed of a silica-rich matrix that likely consists of opal and cristobalite and within which a diverse range of particulates is embedded (Fig. 3C,D). Particulates are variously composed of minerals such as detrital quartz, hematite, muscovite, clinochlore, amphiboles, and feldspars^20^, and the presence of high-temperature spinels such as magnetite and silicates such as olivine and pyroxene is expected^52–54^. Metal sulfate layers are typically present along coating surfaces, within coatings, or at interfaces between coatings and underlying rock; and these layers contain minerals such as jarosite, plumbojarosite, and lead-rich members of the alunite group. Overall, rock coatings in the Rouyn-Noranda region are enriched in elements such as iron, arsenic, tin, lead, copper, and zinc^20^. Boundaries between silica-rich layers and underlying rock are generally sharp, which suggests that the coatings formed through an evaporation-dissolution-reprecipitation process^8,10,20,55^.

Coating thicknesses in the Rouyn-Noranda region are generally a function of both rock composition and distance from the Horne smelter. In particular, coatings tend to be thickest on mafic substrates, and are mostly limited to smelter distances of less than ~6 km^20^. Since elevated sulfur concentrations are known to have extended far beyond this distance^48,56^, the controlling factor for coating development here has likely been the greater deposition of metal-bearing particulate matter at shorter smelter distances^20^. Over timespans of decades, rock coatings in the study area are resistant to physical and chemical weathering, and coatings have thus generally remained well preserved in the study area. However, localized physical weathering of coatings has taken place in areas subjected to relatively high levels of pedestrian traffic, and some additional loss of coatings has taken place as a result of the removal or burial of coated bedrock during landscaping and the construction of new buildings and infrastructure (Fig. 3B).

### Collection of Reflectance Spectra of Rock Coatings in the Rouyn-Noranda Study Area

**Figure 4:**
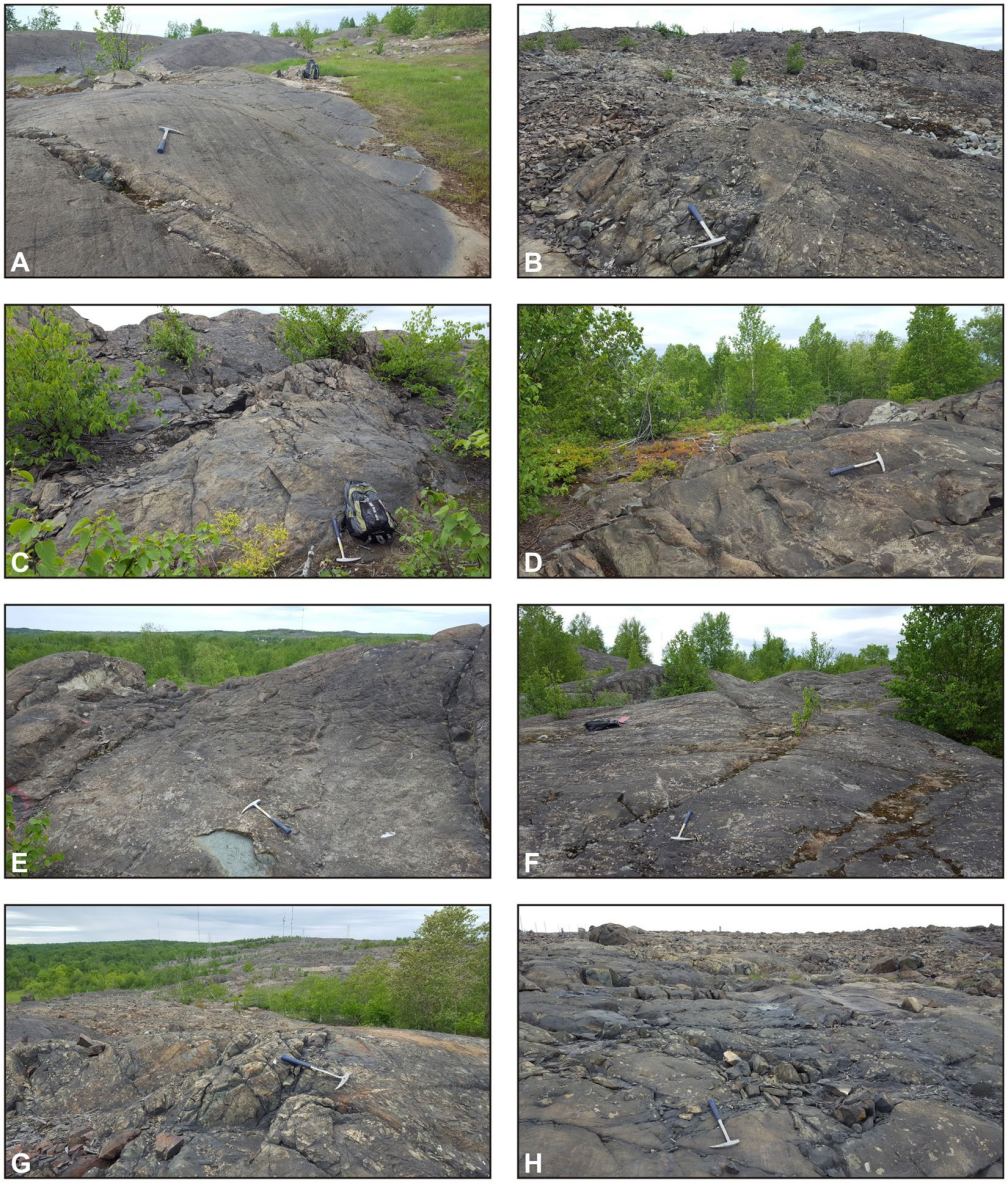
Examples of extensively coated bedrock surfaces near the Horne smelter: (**A**) glacially smoothed and striated igneous units of mafic to intermediate composition, Rouyn-Pelletier formation (Blake River Group) and associated intrusions; (**B**) various intrusive and extrusive igneous units of felsic to intermediate composition; (**C**) rhyolite of the Noranda formation (Blake River Group); (**D**–**F**) basaltic and andesitic lava flows of the Rouyn-Pelletier formation; (**G**,**H**) Quémont and Joliet rhyolites (Blake River Group). Rock hammer for scale. Site locations are given in Fig. 2.

**Figure 5:**
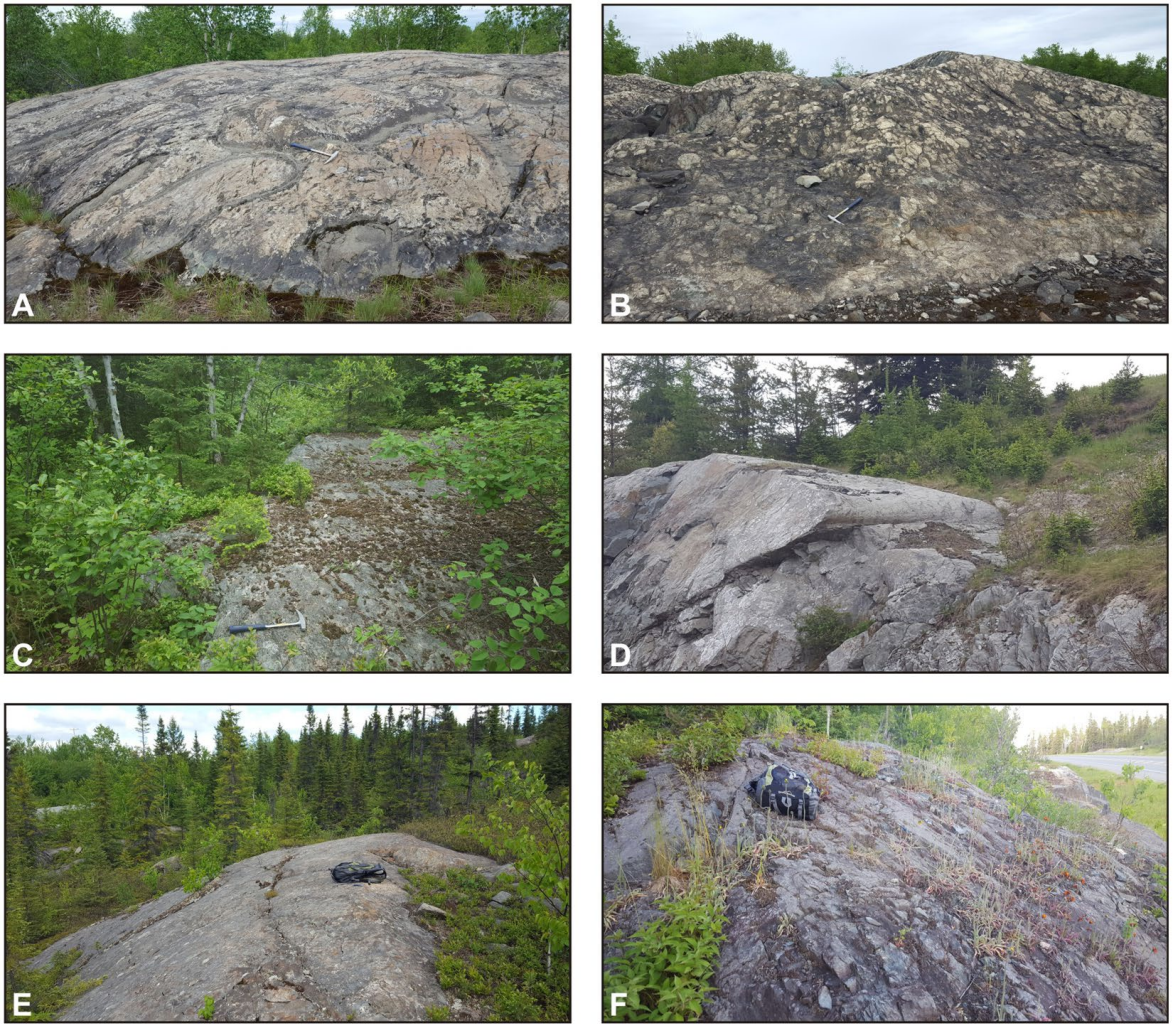
Examples of partly coated (**A**,**B**) and uncoated (**C**–**F**) bedrock surfaces in the Rouyn-Noranda study area: (**A**) glacially smoothed basaltic and andesitic pillow lavas of the Rouyn-Pelletier formation (Blake River Group); (**B**) rhyolite breccia consisting of angular fragments set in a hyaloclastite matrix (Blake River Group); (**C**) sediments of the Granada Formation (Timiskaming Group); (**D**) mafic and intermediate units of the Rouyn-Pelletier formation (Blake River Group) and a younger gabbro intrusion; (**E**) tonalite of the D’Alembert pluton; (**F**) tonalite of the Powell pluton. Rock hammer and daypack for scale. Site locations are given in Fig. 2.

Samples of common types of bedrock in the Rouyn-Noranda region were collected in the study area, with a special emphasis on materials located within ~10 km of the Horne smelter. Coated rock exposures here range from those characterized by prominent and extensive coatings (e.g., Fig. 4) to those with less conspicuous coatings that discontinuously extend across rock surfaces (e.g., Fig. 5A,B). Consistent with a recent field survey^20^, distinct rock coatings were not observed in the study area at smelter distances greater than ~6 km (e.g., Fig. 5C–F).

The reflectance spectra of uncoated and coated surfaces of selected rock samples collected within 6 km of the Horne smelter were determined in a controlled lab setting using an Analytical Spectral Devices FieldSpec3 spectroradiometer equipped with a contact probe. The spectral resolution of this system is ~3–12 nm and varies with wavelength. A white Lambertian reference panel was used to calibrate the system on the basis of standard techniques, allowing for the calculation of reflectance ratios based on the electromagnetic radiation reflected off of samples and the reference panel under identical illumination conditions^57^.

Reflectance spectra were used to describe the manner in which coatings in the Rouyn-Noranda region reflect incoming radiation at visible, near-infrared, and shortwave infrared wavelengths. Notable associated absorption features were identified and were related to the mineralogical and elemental components of coatings. Comparisons between Rouyn-Noranda spectra and those previously collected in the Sudbury region were performed in order to determine if notable differences exist between the reflectance properties of coatings produced by emissions from the Horne smelter and those of coatings produced in the Sudbury region by the Falconbridge, Coniston, and Copper Cliff smelters^11,12^.

### Satellite Data and Classification Methodology

Landsat multispectral data have spectral and spatial characteristics that are well suited for surface cover mapping at local and regional scales and for the successful discrimination of a variety of geological classes^58–62^. Archived Landsat images are currently available at no cost to users, providing universal access to a large collection of multispectral image databases with extensive geographic and temporal coverage.

**Figure 6:**
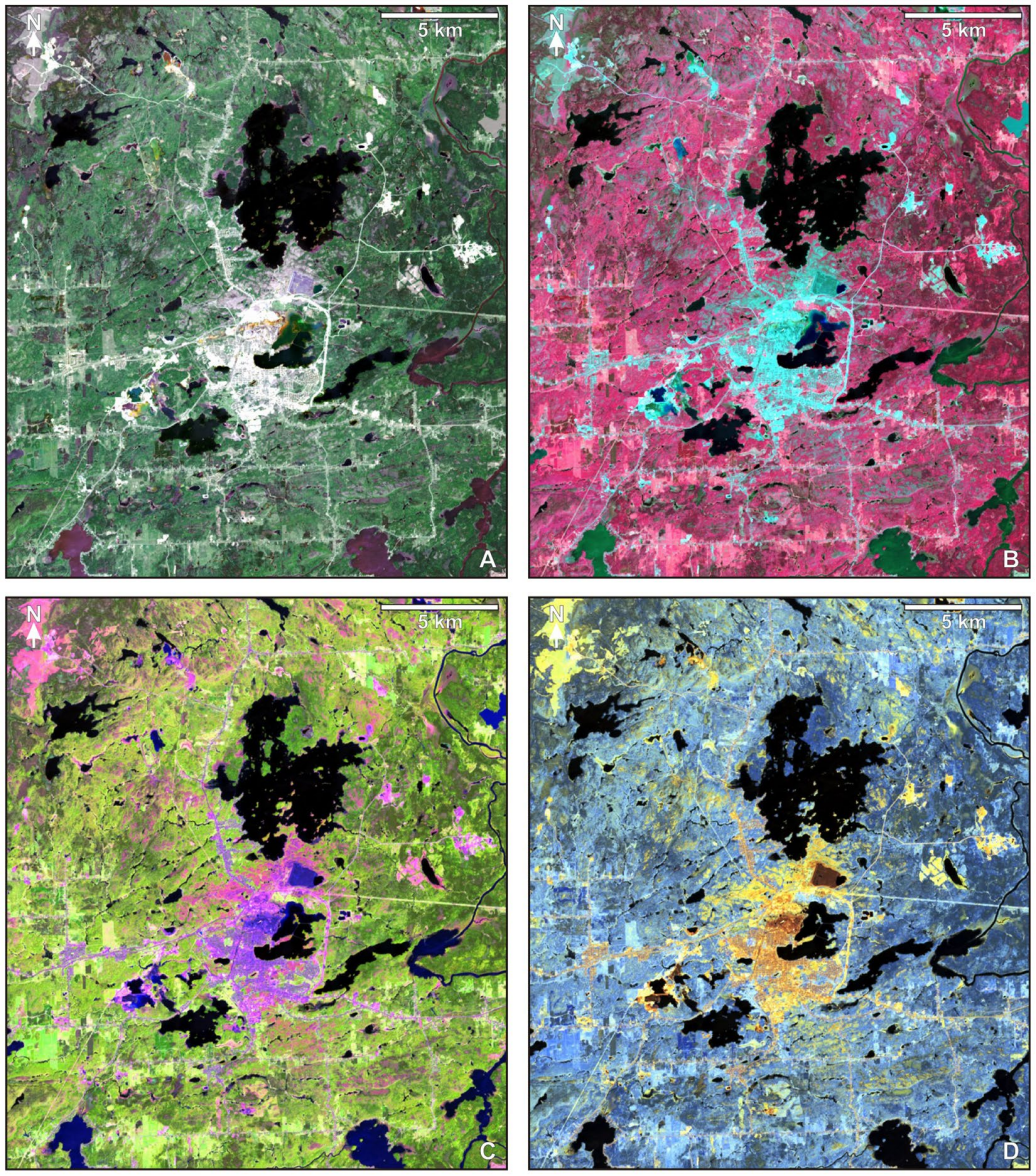
Color composites of Landsat 8 OLI reflectance data for the Rouyn-Noranda study area (Fig. 2): (**A**) OLI 432-RGB (true color); (**B**) OLI 543-RGB (color infrared); (**C**) OLI 654-RGB; (**D**) OLI 765-RGB. Urbanized parts of Rouyn-Noranda are located mainly at lower center. More generally, areas where ground materials of various types are relatively well exposed are highlighted in shades of yellow in “D”. Image processing was performed using Geomatica software^78^.

This study involved the supervised classification of a surface reflectance database derived from orthorectified and radiometrically calibrated Landsat 8 Operational Land Imager (OLI) data (Fig. 6). Reflectance data were produced by the United States Geological Survey from a cloud-free image acquired for the Rouyn-Noranda region on June 8, 2017 (Landsat Product ID: LC08_L1TP_018026_20170608_20170616_01_T1) using the Landsat 8 Surface Reflectance Code (LaSRC). Utilized reflectance data were derived from OLI bands 2 to 7, covering wavelength ranges within the blue, green, red, near-infrared, and shortwave infrared (Band 2: 452–512 nm; Band 3: 533–590 nm; Band 4: 636–673 nm; Band 5: 851–879 nm; Band 6: 1566–1651 nm; Band 7: 2107–2294 nm). The reflectance database has a uniform pixel size of 30 × 30 m.

Prior research demonstrated the effectiveness of the maximum likelihood algorithm in the remote-sensing-based identification of coated rock surfaces in the Sudbury region of Ontario, Canada^11,12^. In that work, the maximum likelihood algorithm, with null class implemented, outperformed a feedforward backpropagation neural network classifier and the Spectral Angle Mapper classifier by consistently producing useful and representative coating maps characterized by low proportions of false positives. Using the Sudbury results as a basis, the present study focused on classification of the Landsat 8 reflectance database using the maximum likelihood classifier.

Among the most widely used routines for supervised classification, the maximum likelihood algorithm parameterizes the ranges of satellite image values that characterize the training sites of particular classes using Gaussian probability distributions derived from associated mean vectors and matrices of covariance^63^. For the utilized version of the maximum likelihood algorithm, the *a priori* probability of the presence of particular surface classes^64^ is assumed to be equal for all classes, and individual pixel labels are assigned to the classes that have the highest associated probabilities. The use of a null class ensures that pixels are only labeled if peak probabilities exceed a threshold, which in the utilized algorithm is defined by a hyperellipsoid with a surface located three standard deviations from the mean vector of any given class. This can improve classification outcomes for e.g. the rock coating class by reducing false positive detections in areas where materials with somewhat similar reflectance characteristics are present (e.g., mine tailings)^11,12^. Though rock coatings were of prime interest in this study, the utilized algorithm requires the specification of a representative and complete list of general surface cover classes. Classifications were produced for the Rouyn-Noranda study area using the following set of classes: coated rock, uncoated rock, uncoated urban materials, uncoated clearings and open pits, predominantly coniferous vegetated sites, predominately deciduous or grassy vegetated sites, deeper water, and shallower or turbid water.

Classification of the Landsat 8 reflectance database allowed for the generation of a map of exposed rock coatings for the Rouyn-Noranda study area. Predicted sites of extensive rock coatings were evaluated in terms of their qualitative consistency with field-based knowledge of the study area gained in this study and in a previous study^20^. Quantitative evaluations of surface cover data were also performed, based on comparison of classification outcomes with test pixels known to be dominated by particular surface cover classes. The numbers of test pixels used in this study were as follows: coated rock (180 test pixels), uncoated rock (288 test pixels), uncoated urban materials (519 test pixels), uncoated clearings and open pits (434 test pixels), predominantly coniferous vegetated sites (349 test pixels), predominantly deciduous vegetated sites (391 test pixels), deeper water (168 test pixels) and shallower or turbid water (255 test pixels).

## Results

Results generated in this study, including those related to both the spectral characterization of Rouyn-Noranda rock coatings and the generation of a map of prominent rock coatings in the study area, are given below.

### Reflectance Properties of Coatings in the Rouyn-Noranda Study Area

**Figure 7:**
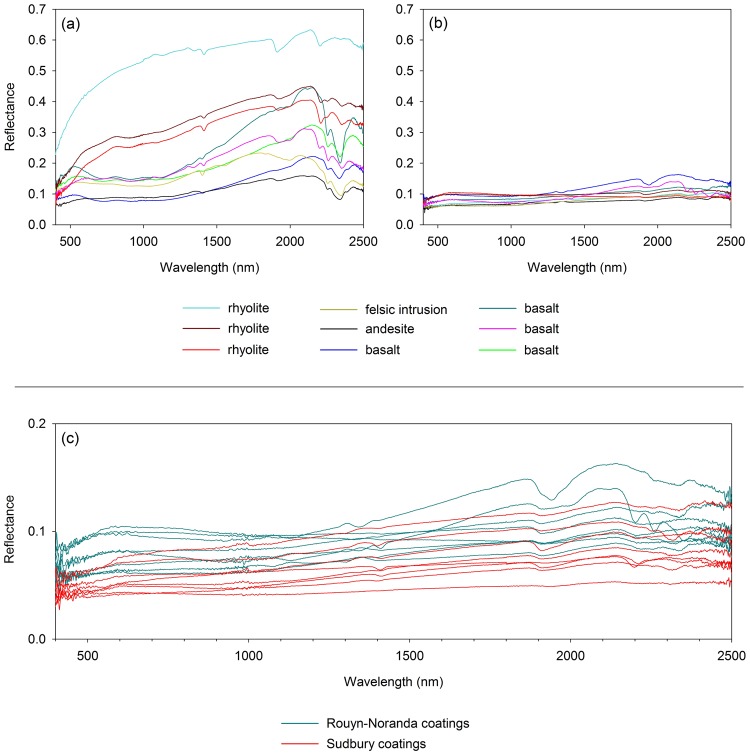
(**a**,**b**) Reflectance spectra of uncoated (**a**) and corresponding coated (**b**) bedrock surfaces within 6 km of the Horne smelter, for lithologies common in the Rouyn-Noranda study area. As is typical of rock coatings formed near smelters^11,12^, coatings in the Rouyn-Noranda study area are effective in masking the reflectance properties of underlying lithologies. (**c**) Comparison between reflectance spectra of rock coatings from the Rouyn-Noranda study area (teal) and from Sudbury (red)^11^. Though Rouyn-Noranda coatings can have slightly higher reflectance values in the visible and shortwave infrared than Sudbury coatings, the overall reflectance properties of coatings from both sites are similar.

Reflectance spectra of selected coated and uncoated rock surfaces from the Rouyn-Noranda study area are given in Fig. 7a,b. Though typically less than 50 micrometers thick, coatings are generally very effective at masking the spectral characteristics of the underlying rocks. Overall, coated materials have relatively low albedo across the visible, near-infrared, and shortwave infrared. The spectra of coatings are somewhat flat across this range, with typical reflectance values collectively ranging mainly between lows of ~5% at shorter wavelengths and highs of ~16% in the shortwave infrared.

The reflectance spectra of rock coatings in the Rouyn-Noranda study area are characterized by relatively subtle absorption features within the visible-to-shortwave-infrared range. Broad and shallow absorption features near 1000 nm suggest the presence of iron-bearing minerals such as olivine and pyroxene^65,66^, which are high-temperature minerals expected to comprise some smelter-derived spheroidal clasts embedded within the silica-rich matrix of coatings. Absorption features near 1400 and 1900 nm suggest the presence of water^67^, which is consistent with the expected presence of constituents such as opaline silica as well as hydrous phyllosilicates and sulfates. Features near 2200 and 2350 nm are consistent with the presence of OH-bearing minerals including clays, iron hydroxides, and iron sulfates^67–69^. Absorption near 2350 nm is also consistent with the presence of carbonates^70^, but minerals of this class are not expected in significant amounts due to the relatively acidic conditions that were present during coating formation. Absorption features such as those near 900, 2200, 2260, 2325, and 2350 nm are consistent with the presence of minerals including sulfates^71,72^, which are confirmed constituents of some coating surfaces and can also be present within coatings and along the interfaces between coatings and underlying substrates (Fig. 3C,D).

The visible-to-shortwave-infrared reflectance properties of rock coatings in the Rouyn-Noranda study area are very similar to those previously determined for rock coatings in the Sudbury region (Fig. 7c)^11,12^, though there is a tendency for some Rouyn-Noranda coatings to have slightly higher reflectance values in the visible and shortwave-infrared ranges than their Sudbury counterparts. Overall, coatings from both Sudbury and Rouyn-Noranda have relatively low reflectance values that increase slightly from the visible into the adjacent infrared. There a close correspondence between absorption features displayed in both sets of spectra, and this correspondence is consistent with the comparable mineralogical and chemical compositions of coatings from these two areas^20^.

Magnetite and carbon-rich particles are typical products of combustion processes^73,74^, and should be common components of coatings near both Sudbury and Rouyn-Noranda^8,20^. These phases commonly generate visible-to-shortwave-infrared spectra of low albedo with few absorption features^75,76^ and may play a role in determining the overall reflectance characteristics of coatings near these two smelting centers^11,12^.

### Discrimination of Rock Coatings Using Landsat 8 Reflectance Data

**Figure 8:**
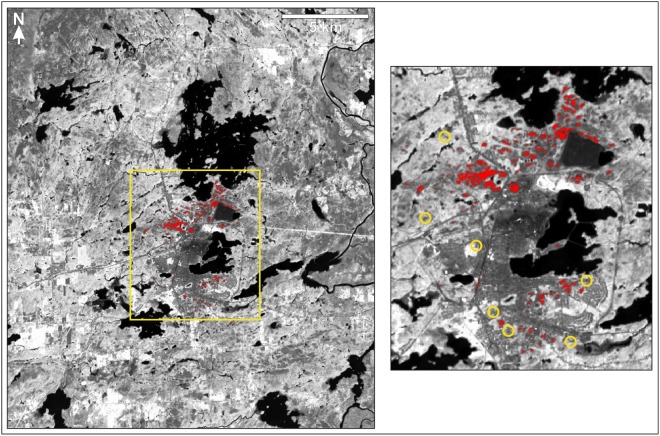
Spatial distribution of the most prominently coated bedrock exposures (red) in the Rouyn-Noranda study area (Fig. 2), as generated by a maximum likelihood classifier with a null class (overlain on reflectance data derived from OLI Band 5, acquired in the near infrared). This database successfully identifies areas that are notably characterized by the presence of distinct coatings, with a low proportion of false positives, and provides a good overview of the general spatial distribution of coated bedrock in the Rouyn-Noranda study area. However, the database does not fully identify areas containing less distinctly or less extensively coated bedrock; selected sites where partially coated bedrock exists but was not discriminated by the classifier are identified by yellow circles. Image at right is an enlargement of the area outlined in yellow at left. Image classification and processing were performed using Geomatica software^78^.

Maximum-likelihood classification of the Landsat 8 reflectance database allowed for the generation of a map of extensively and prominently coated bedrock (Fig. 8) that is qualitatively consistent with field experience related to this study and other recent work^20^. In particular, areas of notable bedrock coatings (e.g., Fig. 4) are confirmed to be restricted to smelter distances of less than ~6 kilometers. Prominent coatings are especially concentrated in areas located immediately west, north, and northeast of the Horne smelter. Other areas of notable coating exposure include those located south of Lac Osisko (Figs 2 and 8). In the generated map of coated bedrock, exposures of uncoated bedrock and other uncoated materials (depicted as dark-yellow pixels in Fig. 6D) are appropriately not labeled as coated. However, the map of coated bedrock problematically does not fully identify areas that are less distinctly coated (e.g., yellow circled areas in Fig. 8), resulting in the underestimation of the total spatial extent of coated or partly coated bedrock. This restricts the map’s utility primarily to the highlighting of the most extensively and thickly coated bedrock exposures in the study area, and to the delineation of areas that were especially affected by past emissions of the Horne smelter.

Overall, 141 of 180 coated bedrock test pixels (78.3%) were correctly identified as such, 19 of 180 coated bedrock test pixels (10.5%) were not classified and were instead assigned to the null class, and only 20 of 180 coated bedrock test pixels (11.1%) were misclassified as uncoated (with 12 of 180 misclassified as uncoated bedrock and 8 of 180 misclassified as uncoated urban) (Table 1). These relatively minor levels of misclassification are not unexpected, as there is a continuum of coating extent (from 100% cover to negligible or absent cover) that necessarily requires that some areas described as coated by observers on the ground may be insufficiently distinct to be properly identified as coated by a classifier working with relatively large pixels (30 × 30 m). Misclassified coated pixels are associated with materials with reflectance properties that are either similar to those of coatings (e.g., some uncoated urban materials) or that grade from coated bedrock to other related classes (e.g., uncoated bedrock). In the classification of Landsat 8 reflectance data, none of the test pixels for the seven other classes were misclassified as coated, confirming that false positives are uncommon for the coated bedrock class.

**Table 1:** Summary of maximum likelihood results for classifications with a null class (A) and without a null class (B).

*coated rock correctly classified*	*coated rock misclassified as uncoated rock*	*coated rock misclassified as uncoated urban*	*coated rock assigned to null class*
**(A) With Null Class**			
78.3%	6.7%	4.4%	10.5%
**(B) Without Null Class**			
90.5%	7.2%	2.2%	n/a

Classes mislabelled as coated bedrock for the classification with a null class: none. Classes mislabelled as coated bedrock for the classification without a null class: uncoated bedrock (4.9%), shallower water (0.4%), uncoated urban 0.2%). Though the classification without a null class has a higher proportion of coated bedrock test pixels that were correctly classified as such, it is also characterized by problematic false positives involving other classes, most notably the uncoated bedrock class.

**Figure 9:**
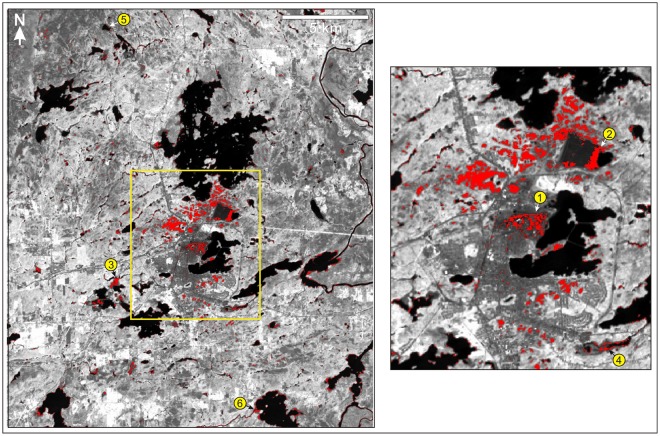
Spatial distribution of coated bedrock exposures (red) in the Rouyn-Noranda study area (Fig. 2), as generated by a maximum likelihood classifier without a null class (overlain on reflectance data derived from OLI Band 5, acquired in the near infrared); compare with Fig. 8. Though this database expands the successful detection of coated bedrock in the study area relative to that of the database given in Fig. 8, it is also characterized by numerous examples of false positives, rendering the database much less useful. Some of the most notable examples of false positives are labeled with numbered yellow circles: (1) geological materials exposed within the Horne smelter site; (2 and 3) geological deposits in tailings ponds; (4) areas of exposed uncoated rock and organic materials in wetlands; (5 and 6) uncoated rock exposed or very shallowly submerged along lake shorelines. Image at right is an enlargement of the area outlined in yellow at left. Image classification and processing were performed using Geomatica software^78^.

A higher proportion of coated or partly coated exposures of bedrock was successfully identified in a maximum likelihood classification performed without the null class (Fig. 9). Specifically, 163 of 180 coated bedrock test pixels (90.5%) were correctly identified as such, and 17 of 180 coated bedrock test pixels (9.4%) misclassified as uncoated (with 13 of 180 misclassified as uncoated bedrock and 4 of 180 misclassified as uncoated urban) (Table 1). However, the absence of a null class allowed pixels to be labeled without a minimum probability threshold, which permitted labels to be assigned under circumstances in which single classes were not strongly favored with high estimated probabilities. In the generated database, confusion between the coated bedrock class and spectrally similar classes (e.g., geological materials in tailings ponds) was correspondingly increased, and the proportion of false positives for the coated bedrock class was also increased, reducing the utility of this database relative to that generated through the implementation of a null class. Though only 1 of 255 shallow water test pixels (0.4%) and 1 of 519 uncoated urban test pixels (0.2%) were misclassified as coated bedrock in the classification conducted without a null class, 14 of 288 uncoated bedrock test pixels (4.9%) were more problematically misclassified as coated bedrock, underscoring the reduced confidence with which the associated map can be used to identify coated sites in the Rouyn-Noranda study area. False positives can be especially problematic since they can in some instances wrongly suggest that coated bedrock is present in areas where no coatings exist at all, whereas false negatives have a tendency to nevertheless be proximal to sites correctly identified as coated, and thus tend to have much less of a negative impact on the overall visual impression of the predicted spatial distribution of coatings.

## Discussion

The reflectance spectra of Rouyn-Noranda rock coatings have properties that are comparable to those previously measured in the Sudbury region, which is consistent with associated similarities in mineralogical and elemental compositions. Coatings near both Rouyn-Noranda and Sudbury tend to have relatively flat reflectance spectra, involving overall reflectance values of roughly 5 to 15% across the visible, near-infrared, and shortwave infrared. Both sets of spectra are characterized by subtle absorption features consistent with the presence of known or likely components including opaline silica, hydrous clays, sulfates, carbon-rich particulates, and high-temperature oxides and silicates. Though notable similarities can exist between the spectra of rock coatings and those of materials including mine tailings^12^, the most prominently coated bedrock surfaces in the Rouyn-Noranda study area were successfully discriminated from other surface classes through the supervised classification of reflectance data derived from Landsat OLI data. Less prominently coated surfaces were not identified in all cases. As previously determined for the Sudbury region, the use of a null class greatly reduces the occurrence of false positives in maps depicting the spatial distribution of coated rock, improving the utility of associated databases despite the lower proportion of correctly identified sites of coated bedrock.

The mapped extent of prominent rock coatings depicted in Fig. 8 is representative of the time frame in which image data were acquired (i.e., June, 2017), and is limited to sites where coatings remained well preserved and well exposed at that time. The mapped extent of prominent coatings generated using the Landsat 8 reflectance database does not include areas where coatings may have once existed but later became extensively weathered, vegetated, or buried (e.g., Fig. 3B). The mapped extent of coatings also does not include areas where the effects of smelter emissions may have been severe but where the absence of an appropriate rock substrate prevented the formation of coatings (e.g., areas mantled by glaciofluvial sediments). Given these considerations, the mapped extent of coatings should mainly be utilized as a general guide regarding the approximate smelter distances within which the combined effects of acid rain and the deposition of smelter particulates were greatest.

A total of 1287 pixel locations are predicted to have been associated with well-exposed fully or partly coated bedrock in the Rouyn-Noranda study area in 2017. With pixel areas of 30 × 30 m (=900 m^2^), this gives a total predicted surface area of 1,158,300 m^2^, or ~1.2 km^2^. A classification success rate of 78% for the coated bedrock class (Section 7.2) implies a current total surface area of rock coatings of ~1.5 km^2^. Though notable, the spatial extent of prominent dark coatings in the Rouyn-Noranda study area is only roughly 1% of that which characterizes the Sudbury region^11^, where emissions from historical roast yards and three major smelting centers prior to the mid-1970s produced a total surface area of exposed rock coatings that is estimated for the year 2000 to have been as great as ~155 km^2^.

Lichen cover can be significant on bedrock in the Rouyn-Noranda study area, and, where extensive, can reduce the effectiveness of remote sensing techniques in the successful discrimination of associated geological classes^41^. However, there is a general tendency in the study area for coated bedrock surfaces to be characterized by much lower amounts of lichen cover than uncoated surfaces. Since the reflectance characteristics of lichens can differ considerably from those of bedrock^41,59,77^, the greater tendency for lichens to colonize uncoated bedrock may assist in the discrimination of coated bedrock from uncoated bedrock by further increasing the spectral separation between these classes.

This study utilized Landsat 8 data characterized by a relatively coarse pixel size of 30 × 30 m. Improved outcomes in the discrimination of rock coatings may be possible, especially where coatings are notably discontinuous and irregularly distributed, through the use of image databases with higher spatial resolutions. In such areas, smaller pixel sizes may increase the abundance of relatively pure pixels (i.e., individual pixel locations dominated by single classes), possibly providing a clearer basis for discrimination of the coated class. Improved classification outcomes may also result from the use of hyperspectral rather than multispectral image databases, since differences in the reflectance properties of the coated class and certain uncoated classes (e.g., mine tailings and asphalt^11,12^) can be subtle and are less likely to be consistently detectable using image databases with poorer spectral characteristics.

Classification results for the Rouyn-Noranda study area confirm that multispectral datasets can be used to detect and map prominent rock coatings near smelters, highlighting the overall spatial distribution of sites previously subjected to severe levels of acid rain and smelter-derived particulates. Remote sensing techniques should be applicable to the study of the spatial distributions of rock coatings at smelting sites beyond those of Sudbury and Rouyn-Noranda, including sites where environmental conditions are sufficiently severe for the development of rock coatings to be ongoing. For example, a time series of maps of rock coatings could potentially be used to better understand the spatial distribution of ongoing environmental degradation in the vicinities of smelters that are actively emitting high levels of particulates, heavy metals, and sulfur dioxide. Remote sensing techniques may similarly prove useful in the monitoring of environmental recovery where smelter emissions have been reduced or eliminated, by facilitating the generation of maps that depict the progressive reduction of exposed coatings as a result of weathering and erosion, revegetation, and the construction of roads and other infrastructure.

## Conclusions

1. Prior to the mid-1970s, development of rock coatings was promoted in the Rouyn-Noranda region by emissions of particulates and sulfur dioxide from the Horne smelter. Though typically less than 50 micrometers thick, these coatings are generally very effective at masking the reflectance characteristics of rock substrates.
2. Overall, coatings are similar in composition to those previously investigated in the Sudbury region, and correspondingly have similar spectral characteristics. Rock coatings in the Rouyn-Noranda region have relatively low albedo across the visible, near-infrared, and shortwave infrared, and associated reflectance spectra are characterized by subtle absorption features that are consistent with the known or expected presence of constituents including opaline silica, olivine, pyroxene, hydrous phyllosilicates, and sulfates.
3. Classification of a reflectance database derived from Landsat 8 OLI data successfully produced a map of prominently coated bedrock in the Rouyn-Noranda region. This map does not identify all areas that are less distinctly coated, and thus underestimates the total spatial extent of coated bedrock here. Detection of additional exposures of coated bedrock could not be performed without increasing confusion between coated bedrock and uncoated materials such as mine tailings.
4. The total surface area of rock coatings near Rouyn-Noranda is estimated to currently be ~1.5 km^2^, nearly all of which is located within ~6 km of the Horne smelter.
5. Improved outcomes in the discrimination between rock coatings and other classes may be possible through the use of image databases with higher spatial resolutions and/or enhanced spectral characteristics.

## References

[1] Dudka S, Adriano DC (1995). Environmental impacts of metal ore mining and processing: A review. J. Environ. Qual..

[2] Mudd GM (2010). Global trends and environmental issues in nickel mining: Sulfides versus laterites. Ore Geol. Rev..

[3] Freedman B, Hutchinson TC (1980). Pollutant inputs from the atmosphere and accumulations in soils and vegetation near a nickel-copper smelter at Sudbury, Ontario, Canada. Can. J. Botany.

[4] Adamo P, Dudka S, Wilson MJ, McHardy WJ (1996). Chemical and mineralogical forms of Cu and Ni in contaminated soils from the Sudbury mining and smelting region, Canada. Environ. Pollut..

[5] Freedman B, Hutchinson TC (1980). Long-term effects of smelter pollution at Sudbury, Ontario, on forest community composition. Can. J. Botany.

[6] Winterhalder, K., The effects of the mining and smelting industry on Sudbury’s landscape. In *The Physical Environment of the City of Greater Sudbury*, edited by Rousell, D. H. & Jansons, K. J., 145–173, Special Volume 6, Sudbury, Ontario Geological Survey (2002).

[7] Schindler M, Durocher J, Abdu Y, Hawthorne FC (2009). Hydrous silica coatings: Occurrence, speciation of metals, and environmental significance. Environ. Sci. Technol..

[8] Mantha NM, Schindler M, Murayama M, Hochella MF (2012). Silica- and sulfate-bearing rock coatings in smelter areas: products of chemical weathering and atmospheric pollution. I. Formation and mineralogical composition. Geochim. Cosmochim. Ac..

[9] Mantha NM, Schindler M, Kyser TK (2012). Silica- and sulfate-bearing rock coatings in smelter areas: part II. Forensic tools for atmospheric metal(loid)- and sulfur-isotope compositions. Geochim. Cosmochim. Ac..

[10] Schindler M, Mantha N, Kyser KT, Murayama M, Hochella MF (2012). Shining light on black rock coatings in smelter-impacted areas. Geosci. Can..

[11] Malcolm KJ, Leverington DW, Schindler M (2015). A Landsat-based study of black rock coatings proximal to base metal smelters, Sudbury, Ontario, Canada. Int. J. Remote Sens..

[12] Leverington DW, Schindler M (2016). Detection and mapping of black rock coatings using Hyperion images: Sudbury, Ontario, Canada. Remote Sens..

[13] Kettles IM, Bonham-Carter GF (2002). Modelling dispersal of metals from a copper smelter at Rouyn-Noranda (Québec, Canada) using peatland data. Geochem-Explor. Env. A..

[14] Savard MM, Bégin C (2004). Parent, Michel, Smirnoff, A., & Marion, J. Effects of smelter sulfur dioxide emissions: a spatiotemporal perspective using carbon isotopes in tree rings. J. Environ. Qual..

[15] Glooschenko WA, Holloway L, Arafat N (1986). The use of mires in monitoring the atmospheric deposition of heavy metals. Aquat. Bot..

[16] Watmough SA, Hutchinson TC (1997). Metal resistance in red maple (Acer rubrum) callus cultures from mine and smelter sites in Canada. Can. J. Forest Res..

[17] Dumontet S, Dinel H, Lévesque PEN (1992). The distribution of pollutant heavy metals and their effect on soil respiration and acid phosphatase activity in mineral soils of the Rouyn-Noranda region, Québec. Sci. Total Environ..

[18] Doyle PJ (2003). An ecological risk assessment of air emissions of trace metals from copper and zinc production facilities. Hum. Ecol. Risk Assess..

[19] Masson S (2010). Responses of two sentinel species (*Hexagenia libata* – mayfly; *Pyganodon grandis* – bivalve) along spatial cadmium gradients in lakes and rivers in northwestern Québec. J. Environ. Monitor..

[20] Caplette JN, Schindler M, Kyser TK (2015). The black rock coatings in Rouyn-Noranda, Québec: fingerprints of historical smelter emissions and the local ore. Can. J. Earth Sci..

[21] Ayer J (2002). Evolution of the southern Abitibi greenstone belt based on U-Pb geochronology: autochthonous volcanic construction followed by plutonism, regional deformation and sedimentation. Precambrian Res..

[22] Thurston PC, Ayer JA, Goutier J, Hamilton MA (2008). Depositional gaps in Abitibi greenstone belt stratigraphy: A key to exploration for syngenetic mineralization. Econ. Geol..

[23] Card, K. D. & Poulsen, K. H. Geology and mineral deposits of the Superior Province of the Canadian Shield. Chapter 2 in *Geology of the Precambrian Superior and Grenville Provinces and Precambrian Fossils in North America*, (co-ord.) S. Lucas; Geological Survey of Canada, Geology of Canada, no. 7, pp.13–194 (1998).

[24] Mercier-Langevin, P. *et al*. *The Blake River Group of the Abitibi Greenstone Belt and its unique VMS and gold-rich VMS endowmen*t. Geological Survey of Canada, Open File 6869, 61 p., Natural Resources Canada, Ottawa (2011).

[25] Powell WG, Carmichael DM, Hodgson CJ (1995). Condition and timing of metamorphism in the southern Abitibi greenstone belt, Quebec. Can. J. Earth Sci..

[26] Ross P-S, Goutier J, Mercier-Langevin P, Dubé B (2011). Basaltic to andesitic volcaniclastic rocks in the Blake River Group, Abitibi Greenstone Belt: 1. Mode of emplacement in three areas. Can. J. Earth Sci..

[27] Lindsey DA (1969). Glacial sedimentology of the Precambrian Gowganda Formation, Ontario, Canada. Geol. Soc. Am. Bull..

[28] Davis DW (2002). U-Pb geochronology of Archean metasedimentary rocks in the Pontiac and Abitibi subprovinces, Quebec, constraints on timing, provenance and regional tectonics. Precambrian Res..

[29] Hannington MD, Santaguida F, Kjarsgaard IM, Cathles LM (2003). Regional-scale hydrothermal alternation in the Central Blake River Group, western Abitibi subprovince, Canada: implications for VMS prospectivity. Miner. Deposita.

[30] Genna D, Gaboury D, Moore L, Mueller WU (2011). Use of micro-XRF chemical analysis for mapping volcanogenic massive sulfide related hydrothermal alteration: Application to the subaqueous felsic dome-flow complex of the Cap d’Ours section, Glenwood rhyolite, Rouyn-Noranda, Québec, Canada. J. Geochem. Explor..

[31] Pearson V, Daigneault R (2009). An Archean megacaldera complex: The Blake River Group, Abitibi greenstone belt. Precambrian Res..

[32] Kerr DJ, Gibson HL (1993). A comparison of the Horne volcanogenic massive sulfide deposit and intracauldron deposits of the Mine sequence, Noranda, Quebec. Econ. Geol..

[33] Mueller WU, Stix J, Corcoran PL, Daigneault R (2009). Subaqueous calderas in the Archean Abitibi greenstone belt: An overview and new ideas. Ore Geol. Rev..

[34] Dimroth E, Imreh L, Rocheleau M, Goulet N (1982). Evolution of the south-central part of the Archean Abitibi Belt, Quebec. Part 1: Stratigraphy and paleogeographic model. Can. J. Earth Sci..

[35] Monecke, T. *et al*. Geology and volcanic setting of the Horne deposit, Rouyn-Noranda, Quebec: initial results of a new research project, Geological Survey of Canada, Current Research, 2008–9, 16 p (2008).

[36] Barrett TJ, MacLean WH, Cattalani S, Hoy L, Riverin G (1991). Massive sulfide deposits of the Noranda area, Quebec. III. The Ansil mine. Can. J. Earth Sci..

[37] National Wetlands Working Group. Wetlands of Canada. Ecological Land Classification Series, No.24, Sustainable Development Branch, Environment Canada, Ottawa, Ontario (1988).

[38] Veillette, J. J., Paradis, S. J. & Buckle, J. Bedrock and surficial geology of the general area around Rouyn-Noranda, Quebec and Ontario. In *Metals in the Environment Around Smelters at Rouyn-Noranda, Quebec, and Belledune, New Brunswick: Results and Conclusions of theGSC MITE Point Sources Project*, (ed.) G. F. Bonham-Carter; Geological Survey of Canada, Bulletin 584, 16 p (2005).

[39] Godbout P-M, Roy M, Veillette JJ, Schaefer JM (2017). Cosmogenic ^10^Be dating of raised shorelines constrains the timing of lake levels in the eastern Lake Agassiz-Ojibway basin. Quaternary Res..

[40] Feisthauer NC, Stephenson GL, Princz JI, Scroggins RP (2006). Effects of metal-contaminated forest soils from the Canadian shield to terrestrial organisms. Environ. Toxicol. Chem..

[41] Rivard B, Zhang J, Feng J, Sanchez-Azofeifa GA (2009). Remote predictive lithological mapping in the Abitibi Greenstone Belt, Canada, using airborne hyperspectral imagery. Can. J. Remote Sens..

[42] Glooschenko WA, Arafat N (1988). Atmospheric deposition of arsenic and selenium across Canada using sphagnum moss as a biomonitor. Sci. Total Environ..

[43] Glooschenko WA (1989). *Sphagnum fuscum* moss as an indicator of atmospheric cadmium deposition across Canada. Environ. Pollut..

[44] Newhook R, Hirtle H, Byrne K, Meek ME (2003). Releases from copper smelters and refineries and zinc plants in Canada: human health exposure and risk characterization. Sci. Total Environ..

[45] Johnson D, Hale B (2004). White birch (*Betula papyrifera* Marshall) foliar litter decomposition in relation to trace metal atmospheric inputs at metal-contaminated and uncontaminated sites near Sudbury, Ontario and Rouyn-Noranda, Quebec, Canada. Environ. Pollut..

[46] Courchesne F, Kruyts N, Legrand P (2006). Labile zinc concentration and free copper ion activity in the rhizosphere of forest soils. Environ. Toxicol. Chem..

[47] Zdanowicz CM, Banic CM, Paktunc DA, Kliza-Petelle DA (2006). Metal emissions from a Cu smelter, Rouyn-Noranda, Québec: characterization of particles sampled in air and snow. Geochem-Explor. Env. A..

[48] Telmer K, Bonham-Carter GF, Kliza D, Hall GEM (2004). The atmospheric transport and deposition of smelter emissions: evidence from the multi-element geochemistry of snow, Quebec, Canada. Geochim. Cosmochim. Acta.

[49] Dixit AS, Alpay S, Dixit SS, Smol JP (2007). Paleolimnological reconstructions of Rouyn-Noranda lakes within the zone of influence of the Horne Smelter, Québec, Canada. J. Paleolimnol..

[50] Hou X (2006). Lead concentrations and isotope ratios in the exchangeable fraction: tracing soil contamination near a copper smelter. Geochem-Explor. Env. A..

[51] Perceval O, Couillard Y, Pinel-Alloul B, Bonneris E, Campbell PGC (2006). Long-term trends in accumulated metals (Cd, Cu and Zn) and metallothionein in bivalves from lakes within a smelter-impacted region. Sci. Total Environ..

[52] Henderson, P. J. & Knight, R. D. Regional distribution and mobility of copper and lead in soils near the Horne copper smelter at Rouyn-Noranda, Quebec. Geological Survey of Canada, Bulletin No. 584 (2005).

[53] Knight RD, Henderson PJ (2006). Smelter dust in humus around Rouyn-Noranda, Quebec. Geochem-Explor. Env. A..

[54] Lanteigne S (2012). Mineralogy and weathering of smelter-derived spherical particles in soils: implications for mobility of Ni and Cu in the surficial environment. Water Air Soil Poll..

[55] Hellmann R, Penisson J-M, Hervig RL, Thomassin J-H, Abrioux M-F (2003). An EFTEM/HRTEM high-resolution study of the near-surface of labradorite feldspar altered at acid pH: evidence for interfacial dissolution-reprecipitation. Phys. Chem. Miner..

[56] Mayer B, Alpay S, Gould WD, Lortie L, Rosa F (2007). The onset of anthropogenic activity recorded in lake sediments in the vicinity of the Horne smelter in Quebec, Canada: sulfur isotope evidence. Appl. Geochem..

[57] Schaepman-Strub G, Schaepman ME, Painter TH, Dangel S, Martonchik JV (2006). Reflectance quantities in optical remote sensing – Definitions and case studies. Remote Sens. Environ..

[58] Leverington DW (2010). Discrimination of sedimentary lithologies using Hyperion and Landsat TM data: A case study at Melville Island, Canadian High Arctic. Int. J. Remote Sens..

[59] Leverington DW, Moon WM (2012). Landsat-TM-based discrimination of lithological units associated with the Purtuniq Ophiolite, Quebec, Canada. Remote Sens..

[60] Adiri Zakaria, Harti Abderrazak El, Jellouli Amine, Maacha Lhou, Bachaoui El Mostafa (2016). Lithological mapping using Landsat 8 OLI and Terra ASTER multispectral data in the Bas Drâa inlier, Moroccan Anti Atlas. Journal of Applied Remote Sensing.

[61] van der Werff H, van der Meer F (2016). Sentinel-2A MSI and Landsat 8 OLI provide data continuity for geological remote sensing. Remote Sens..

[62] Pour Amin Beiranvand, Park Yongcheol, Park Tae-Yoon S., Hong Jong Kuk, Hashim Mazlan, Woo Jusun, Ayoobi Iman (2018). Regional geology mapping using satellite-based remote sensing approach in Northern Victoria Land, Antarctica. Polar Science.

[63] Richards, J. A. *Remote Sensing Digital Image Analysis: An Introduction*. Springer, New York, NY (2012).

[64] Strahler AH (1980). The use of prior probabilities in maximum likelihood classification of remotely sensed data. Remote Sens. Environ..

[65] Adams JB (1968). Lunar and Martian surfaces: Petrologic significance of absorption bands in the near-infrared. Science.

[66] Cloutis E.A., Hiroi T., Gaffey M.J., Alexander C.M.O’D., Mann P. (2011). Spectral reflectance properties of carbonaceous chondrites: 1. CI chondrites. Icarus.

[67] Milliken RE (2008). Opaline silica in young deposits on Mars. Geology.

[68] Hunt GR, Salisbury JW (1970). Visible and near-infrared spectra of minerals and rocks: I. Silicate minerals. Modern Geology.

[69] Sultan M, Arvidson R, Sturchio NC, Guinness EA (1987). Lithologic mapping in arid regions with Landsat Thematic Mapper data: Meatiq Dome, Egypt. Geol. Soc. Am. Bull..

[70] Gaffey SJ (1985). Reflectance spectroscopy in the visible and near-infrared (0.35-2.55 Mm): Applications in carbonate petrology. Geology.

[71] Bishop JL, Murad E (2005). The visible and infrared spectral properties of jarosite and alunite. Am. Mineral..

[72] Cloutis EA (2006). Detection and discrimination of sulfate minerals using reflectance spectroscopy. Icarus.

[73] Gaffney JS, Marley NA (2009). The impacts of combustion emissions on air quality and climate – From coal to biofuels and beyond. Atmos. Environ..

[74] Zajzon N (2013). Integrated mineralogical and magnetic study of magnetic airborne particles from potential pollution sources in industrial-urban environment. Carpath. J. Earth Env..

[75] Cloutis EA, Gaffey MJ, Moslow TF (1994). Spectral reflectance properties of carbon-bearing materials. Icarus.

[76] Scheinost AC, Chavernas A, Barrón V, Torrent J (1998). Use and limitations of second-derivative diffuse reflectance spectroscopy in the visible to near-infrared range to identify and quantify Fe oxide minerals in soils. Clay Clay Miner..

[77] Rivard B, Arvidson RE (1992). Utility of imaging spectrometry for lithologic mapping in Greenland. Photogramm. Eng. Rem. S..

[78] PCI Geomatics, *Geomatica 2012* software package, http://www.pcigeomatics.com/ (2012).

